# Increased Interleukin-36β Expression Promotes Angiogenesis in Japanese Atopic Dermatitis

**DOI:** 10.3390/ijms241311104

**Published:** 2023-07-05

**Authors:** Reo Komaki, Tomomitsu Miyagaki, Miho Tanaka, Kaori Nakajima, Tatsuro Okano, Sora Takeuchi, Takafumi Kadono

**Affiliations:** Department of Dermatology, St. Marianna University School of Medicine, Kawasaki 216-8511, Japan

**Keywords:** atopic dermatitis, IL-36β, vascular endothelial growth factor A, angiogenesis, Asian population

## Abstract

While atopic dermatitis (AD) is considered as a T helper 2 (Th2)-centered disease, an increase in other types of inflammatory cytokines is also noted in AD and they may also contribute to the development of the disease. Recently, the efficacy of an anti-IL-36 receptor antibody in AD was demonstrated in a clinical trial. Although there have been several reports on IL-36α and IL-36γ expression and function in AD, IL-36β has been barely studied. In this report, we examined IL-36β expression and function using clinical samples of AD and the epidermal keratinocyte cell line, HaCaT cells. We demonstrated that IL-36β expression in epidermal keratinocytes was increased in AD lesional skin compared to healthy skin. IL-36β promoted vascular endothelial growth factor A production in HaCaT keratinocytes through phosphorylation of extracellular signal-regulated kinases 1 and 2. In addition, IL-36β up-regulated placental growth factor mRNA expression in HaCaT keratinocytes. IL-36β expression levels in epidermal keratinocytes were correlated with the number of dermal vessels in AD skin. These results suggest that IL-36β may play an important role for angiogenesis in lesional skin of AD and that IL-36β can be a therapeutic target in AD.

## 1. Introduction

Atopic dermatitis (AD) is a common chronic inflammatory skin disease, with an increased prevalence in recent decades [1]. The pathogenesis of AD has been attributed to a complex interaction among immune system abnormalities, barrier impairment, environmental factors, and genetic mutations [1,2,3]. Typically, AD is considered as a T helper 2 (Th2)-dominant disease, characterized by increased IgE production, peripheral eosinophilia, mast cell activation, and induction of Th2 cells expressing various type 2 cytokines. Among type 2 cytokines, IL-4 and IL-13, leading to not only allergic inflammation but also pruritus and barrier dysfunction [3,4,5], are considered to be central in the pathogenesis of AD. Actually, dupilumab, an antibody against IL-4 receptor α, which can block both IL-4 and IL-13 signaling, significantly improved skin lesions and pruritus in patients with moderate to severe AD in two randomized, placebo-controlled clinical trials [6]. The antibody is currently used for AD treatment in clinical settings around the world [7]. In addition to IL-4 and IL-13, several type 2 cytokines, such as IL-31, IL-33, and thymic stromal lymphopoietin, are also considered as therapeutic targets in AD. Nemolizumab, the IL-31 receptor antibody, strongly suppresses the pruritus in AD and is used for AD in some countries [8] and various antibodies to other type 2 cytokines are at various stages of development [9]. Although these antibodies have improved and will improve the quality of life of many AD patients by alleviating symptoms, some patients are resistant to such therapies and other types of inflammatory cytokines may contribute to the development of the disease in these patients. The expression of IL-22, mainly produced by Th17 cells and Th22 cells, is elevated in AD patients [10] and fezakinumab, an antibody to IL-22, was effective for severe AD in the clinical trial [11]. IL-26 produced by Th17 cells was reported to augment type 2 inflammation by inducing IL-33 expression by epidermal keratinocytes [12]. Collectively, AD may be a heterogenous disease, in which various types of cytokines are involved, and therefore exploring the expression and function of inflammatory cytokines is important for deeply understanding the pathogenesis of AD.

The IL-36 cytokine family, belonging to the IL-1 cytokine family, includes three agonists, IL-36α, IL-36β, and IL-36γ, and one antagonist, IL-36 receptor antagonist (IL-36RN), all of which bind to IL-36 receptor (IL-36R) [13,14,15]. Various types of cells in skin, including epidermal keratinocytes, dermal fibroblasts and endothelial cells, as well as several immune cells produce IL-36 cytokines and Th17 cytokines are associated with the production [13,14,15]. IL-36 cytokines have the capacity to augment Th17 immune responses by activation of plasmacytoid dendritic cells and induction of CCL20 from epidermal keratinocytes. They also induce CXCL1, CXCL2, and CXCL8 from epidermal keratinocytes, resulting in neutrophil infiltration in the skin. IL-36 has been revealed to be associated with psoriasis, the representative Th17-dominant disease [16,17]. Recently, emerging evidences suggest that IL-36 cytokines play an important role in the development of AD. The transcriptome analysis using next-generation RNA sequencing first revealed the up-regulation of IL-36 cytokine families in AD lesional skin [18]. Afterwards, increased IL-36α and IL-36γ expression in sera and/or lesional skin in AD patients was confirmed by various groups [19,20,21]. Moreover, recently, the efficacy of an anti-IL-36R antibody, spesolimab, was demonstrated in a multicentre, randomized, double-blind, placebo-controlled study [22]. Thus, IL-36 signaling may play a role in the pathogenesis of AD, whereas the expression and function of IL-36β have been barely studied. In this study, we examined IL-36β expression in sera and lesional skin of AD and investigated its involvement in AD development.

## 2. Results

### 2.1. Increased Expression of IL-36β in Lesional Skin of AD

**Figure 1:**
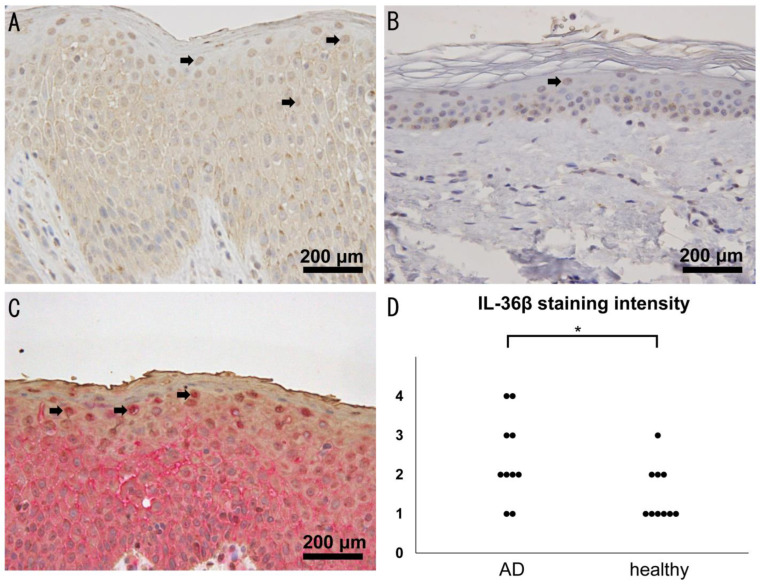
IL-36β is overexpressed on epidermal keratinocytes in lesional skin of atopic dermatitis (AD). (**A**,**B**) IL-36β staining in healthy skin and AD lesional skin (*n* = 10, respectively). Representative images of IL-36β staining in AD skin (**A**) and in healthy skin (**B**) are shown (original magnification ×400). Arrows point to representative keratinocytes with the nuclear staining. (**C**) Double staining of IL-36β (red) and cytokeratin (brown) in AD lesional skin (*n* = 10). Representative image is shown. Arrows point to representative keratinocytes with nuclear IL-36β staining and cytokeratin staining. (**D**) IL-36β staining intensity is shown. The measured values from individual patients are plotted by dots. * *p* **<** 0.05.

To investigate the involvement of IL-36β in AD, we first examined IL-36 expression in lesional skin of AD patients by immunohistochemistry. We found that epidermal keratinocytes mainly expressed IL-36β in both healthy skin and AD skin (Figure 1A,B) similar to psoriasis skin [23]. To clarify whether cells with nuclear IL-36β staining were epidermal keratinocytes or not, we performed double immunohistochemical analysis of IL-36β and cytokeratin in AD skin. Cells with IL-36β staining were positive for cytokeratin (Figure 1C) and considered to be epidermal keratinocytes. In addition, IL-36β staining was observed in part of dermal fibroblasts and vascular endothelial cells (Figure 1A,B). The intensity of IL-36β staining in epidermal keratinocytes was significantly higher in AD skin compared to healthy skin (Figure 1D). We also examined IL-36α, IL-36γ, and IL-36R expression in epidermal keratinocytes of AD by immunohistochemistry and found that IL-36α and IL-36γ expression in epidermal keratinocytes, especially in nucleus, was higher in AD skin (Supplementary Figure S1A–D), consistent with previous studies [19,20,21]. IL-36R expression in epidermal keratinocytes in AD was comparable to that in healthy skin (Supplementary Figure S1E,F). Thus, the expression of the IL-36 cytokine family including IL-36β was increased in AD lesional skin.

### 2.2. Serum IL-36β Levels Are Not Elevated in Patients with AD but Decreased by Dupilumab

**Figure 2:**
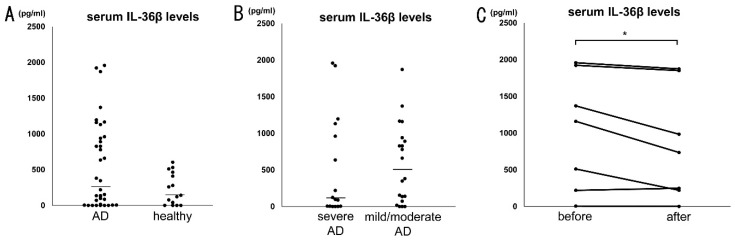
Serum IL-36β levels are not elevated in patients with atopic dermatitis (AD) but decreased by dupilumab. (**A**) Serum IL-36β levels in AD patients (*n* = 36) and healthy control (*n* = 15). (**B**) Serum IL-36β levels in severe AD patients (*n* = 16) and mild/moderate AD patients (*n* = 20). (**C**) Serum IL-36β levels in AD patients (*n* = 7) before and after dupilumab treatment. The measured values from individual patients are plotted by dots. Bars represent the median. * *p* < 0.05.

We also measured serum IL-36β levels in patients with AD and healthy controls. Median serum IL-36β levels in AD patients were 282.9 [13.4–944.4] pg/mL, which tended to be higher than those in healthy controls (141.3 [20.4–437.1] pg/mL), but no statistically significant difference was found between them (*p* = 0.12; Figure 2A). There was also no significant difference in serum IL-36β levels between patients with mild/moderate and severe AD (Figure 2B). Serum IL-36β levels were not correlated with disease severity markers, such as serum lactate dehydrogenase, IgE, and CCL17 levels, eosinophil counts in peripheral blood, and the Eczema Area and Severity Index (EASI) scores. Interestingly, serum IL-36β levels significantly decreased after the dramatical improvement by dupilumab initiation (Figure 2C). Thus, serum IL-36β levels in AD patients were not increased, but dupilumab may have the capacity to diminish IL-36β levels.

### 2.3. IL-36β Promotes VEGF-A and PlGF Expression in HaCaT Cells

**Figure 3:**
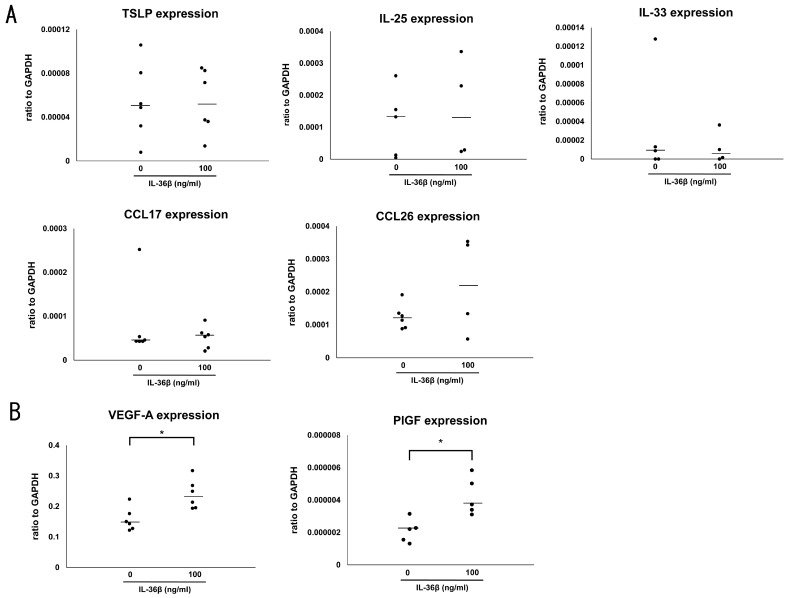
IL-36β promotes vascular endothelial growth factor (VEGF)-A and placental growth factor (PlGF) mRNA expression in HaCaT cells. (**A**,**B**) HaCaT were cultured with recombinant IL-36β (0, 100 ng/mL) for 24 h. Messenger RNA levels of the indicated Th2 cytokines and chemokines (**A**) and angiogenic factors (**B**) were determined by quantitative RT-PCR. One representative result from three independent experiments. The measured values from individual samples are plotted by dots. Bars represent the median. (*n* = 4–6). * *p* < 0.05.

**Figure 4:**
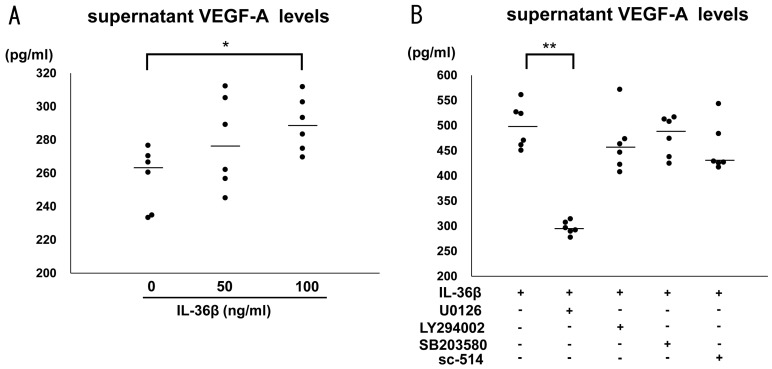
IL-36β enhances vascular endothelial growth factor (VEGF)-A protein production via extracellular signal-regulated kinase (ERK)-1/2 pathway from HaCaT cells. (**A**) HaCaT cells were cultured with recombinant IL-36β (0, 50, 100 ng/mL) for 24 h. Supernatant IL-36β levels were evaluated by enzyme-linked immunosorbent assay. (**B**) HaCaT cells were cultured with recombinant IL-36β (100 ng/mL) for 24 h in the absence or after preincubation with U0126 (20 nM), LY294002 (20 nM), SB203580 (20 nM), or sc-514 (40 nM). Supernatant IL-36β levels were evaluated by enzyme-linked immunosorbent assay. One representative result from three independent experiments. The measured values from individual samples are plotted by dots. Bars represent the median. (*n* = 6). * *p* < 0.05. ** *p* < 0.01.

It is well known that the IL-36 cytokine family induced proinflammatory cytokines and chemokines, such as IL-6, CXCL1, CXCL8, and CCL20 [17,24]. To assess the involvement of IL-36β in AD development, we stimulated HaCaT cells with the recombinant IL-36β protein, and measured levels of various Th2 cytokines and chemokines, and proangiogenic factors, all of which are crucial for the pathogenesis of AD, by quantitative RT-PCR. IL-36β did not induce any Th2 cytokine and chemokines including IL-25, IL-33, thymic stromal lymphopoietin, CCL17, and CCL26 (Figure 3A). Concerning proangiogenic factors, IL-36β augmented both vascular endothelial growth factor (VEGF)-A and placental growth factor (PlGF) mRNA expression from HaCaT cells (Figure 3B). In addition, the induction of VEGF-A protein by IL-36β was also revealed by ELISA using culture supernatants (Figure 4A). Supernatant PlGF protein levels were below the detection limit of ELISA Kit and the increase in protein levels was not confirmed. Thus, IL-36β promoted VEGF-A and PlGF mRNA expression and VEGF-A secretion at protein levels in HaCaT cells.

### 2.4. IL-36β Enhanced VEGF-A Expression via ERK1/2 Pathway in HaCaT Cells

Recently, emerging studies revealed that the IL-36 cytokine family exerts their function through various signaling pathways, such as extracellular signal-regulated kinase (ERK)-1/2 pathway, p38 mitogen activated protein kinase (MAPK) pathway, Akt pathway, and nuclear factor (NF)-κB pathway [25,26]. To investigate which pathway is important for IL-36β-induced VEGF-A protein expression, we precultured HaCaT cells with selective pathway inhibitors, U0126, LY294002, SB203580, and sc-514 followed by stimulation of IL-36β and measured VEGF-A protein expression in the supernatant. Among them, U0126 alone suppressed VEGF-A up-regulation by IL-36β (Figure 4B). Thus, IL-36β induced VEGF-A through ERK pathway in HaCaT cells.

### 2.5. Increased Dermal Vessels in Highly IL-36β-Expressed AD Skin

**Figure 5:**
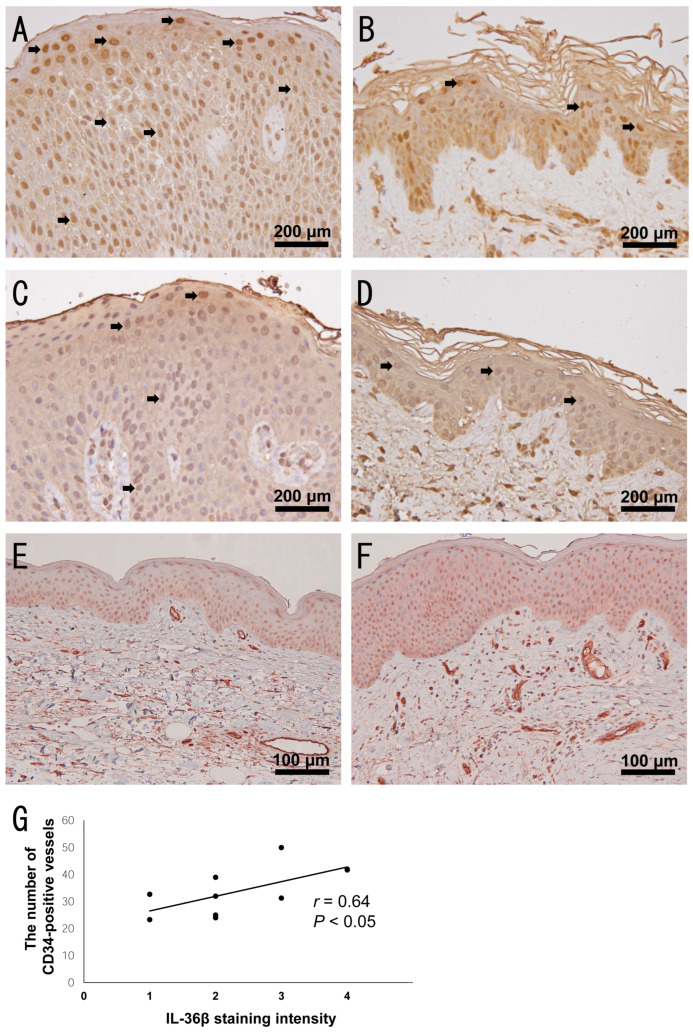
IL-36β staining intensity in epidermal keratinocytes is correlated with the number of dermal vessels in lesional skin with atopic dermatitis (AD). (**A**–**D**) Vascular endothelial growth factor (VEGF)-A (**A**,**B**) and plancental growth factor (PlGF) (**C**,**D**) staining in healthy skin and AD lesional skin (*n* = 10, respectively). Representative images of VEGF-A and PlGF staining in AD skin (**A**,**C**), and in healthy skin (**B**,**D**) are shown (original magnification ×400). Arrows point to representative keratinocytes with the nuclear staining. (**E**,**F**) Double staining of IL-36β (red) and CD34 (brown) in AD lesional skin (*n* = 10). Representative images of AD skin with relatively few dermal vessels (**E**) and with relatively abundant dermal vessels (**F**) are shown (original magnification ×200). (**G**) The correlation between IL-36β staining intensity and the number of CD34-positive dermal vessels (*n* = 10). The measured values from individual patients are plotted by dots.

To investigate the effect of VEGF-A and PlGF induction by IL-36β in clinical samples, we stained AD lesional skin with anti-VEGF-A antibody and anti-PlGF antibody. We found that nuclear staining of VEGF-A in the epidermis was more observed in AD skin compared to healthy skin (Figure 5A,B), whereas PlGF expression in AD skin was comparable to that in healthy skin (Figure 5C,D), consistent with the results of in vitro experiments. These results suggest that IL-36β can induce VEGF-A from epidermal keratinocytes in AD skin, leading to angiogenesis. To clarify that, we performed double immunohistochemical staining of IL-36β and CD34 in AD skin. CD34-positive lumens were more observed in lesional skin with more IL-36β-positive epidermal keratinocytes (Figure 5E,F). The number of CD34-positive lumens in the dermis per low-power field was significantly correlated with the intensity of IL-36β immunostaining in epidermal keratinocytes (Figure 5G). Thus, IL-36β may enhance angiogenesis in AD skin.

## 3. Discussion

Recently, the efficacy of an anti-IL-36R antibody in AD was demonstrated in a clinical trial [22] and IL-36 cytokine family involvement in AD pathogenesis is receiving a lot of attention. In this study, we first showed that IL-36β was expressed mainly by epidermal keratinocytes in lesional skin of AD and that the expression level was higher compared to healthy skin by immunohistochemistry. This result is inconsistent with the result of RNA sequencing using 20 AD lesional skin reported from USA and Europe in 2015 [18]. In the study, mRNA expression of IL-36α, IL-36γ, and IL-36RN but not IL-36β, was up-regulated in AD lesional skin compared to non-lesional skin. Similar results were obtained from the analysis of lesional skin of Tanzanian AD patients [27]. The biggest difference between our study and these studies may be the ethnic background of patients. In our study, all clinical samples were obtained from Japanese subjects. Although AD has common clinical characteristics, such as eczematous lesions and pruritus, a heterogeneous molecular signature with distinct features in different populations has been widely confirmed [28]. Compared to European and American AD, African American AD exhibited attenuated Th1 and Th17 polarization with augmented Th2 and Th22 pathway [29]. On the other hand, Asian AD showed a strong Th17 skewing [30]. IL-36β expression from epidermal keratinocytes was induced mainly by Th17 cytokines, such as IL-17, IL-22, and TNF-α, and the effect of IL-22 was quite modest compared to other two cytokines [16], suggesting that Th17-polarized environment may be important for IL-36β induction in the skin. Actually, IL-36β expression in lesional skin was up-regulated in Th17-skewed diseases, such as psoriasis and hidradenitis suppurativa [31,32]. Collectively, IL-36β expression may be increased in lesional skin of Asian AD patients in which Th17 cytokines are abundant.

We next found that serum IL-36β levels were not increased and correlated with the disease severity markers in AD patients. Consistently, the study from China also reported that IL-36β mRNA expression in the blood was not increased in Asian AD patients [33]. Expression levels of some cytokines, such as IL-17A and IL-26, are reported to be increased in lesional skin but not in the blood, although they contribute to the development of AD [12,34,35]. Thus, IL-36β may also be produced and exert its function mainly in lesional skin similar to those cytokines.

To investigate the function of IL-36β in lesional skin of AD, we stimulated HaCaT keratinocytes with IL-36β and measured the expression of Th2 cytokines, Th2 chemokines, and angiogenic factors. Among them, we found that IL-36β up-regulated VEGF-A mRNA and protein expression in HaCaT cells. Similarly, it has been reported that IL-36α and/or IL-36γ induce VEGF-A from fibroblasts and vascular endothelial cells [36,37]. VEGF-A plays a central role in angiogenesis through inducing endothelial cell proliferation and migration and promoting vessel tube formation, and is associated with the development of various skin inflammatory disorders. In AD, VEGF-A expression levels in serum and skin are elevated and correlated with the disease severity [38]. In addition, an association between VEGF-A gene polymorphism and AD has been reported [39]. Furthermore, VEGF-A inhibitor ameliorates skin inflammation in *APOC1* transgenic mice which develop AD-like skin inflammation spontaneously [40]. Taken together, IL-36β may contribute to AD progression and development through VEGF-A up-regulation and following angiogenesis in lesional skin. Consistently, we found that IL-36β expression levels in epidermal keratinocytes were correlated with the number of dermal vessels in AD skin. In addition to VEGF-A, we newly found that IL-36β up-regulated PlGF mRNA expression in HaCaT cells, although the induction at protein levels was not detected. PlGF belongs to the VEGF family and represents a key regulator of angiogenic events in development and pathologic conditions in skin [41]. PlGF also have the capacity to down-regulate Th1 immune response by modulating the function of dendritic cells [42], resulting in the augmentation of Th2 immune response. PlGF involvement in AD pathogenesis has been barely studied and its importance is unclear, but it was reported that PlGF expression was slightly elevated in epidermal keratinocytes of AD lesional skin [43]. Collectively, PlGF up-regulation by IL-36β might also be associated with the development of AD. It is unclear whether the ability of IL-36β to induce VEGF-A and PlGF expression by epidermal keratinocytes is affected by the ethnic backgrounds and further studies are needed to clarify that.

Interestingly, we found that dupilumab decreased serum IL-36β levels in AD patients. It was reported that dupilumab dampened not only Th2-specific gene expression but also Th17-specific gene expression in lesional skin [44], maybe resulting in decrease in IL-36β expression. Considering our results, dupilumab may suppress angiogenesis induced by up-regulated VEGF-A and PlGF by IL-36β in lesional skin. Actually, the decrease in blood volume and vessel diameter was reported in one dupilumab-treated AD patient [45], whereas investigational studies including many cases are needed.

In summary, IL-36β expression levels were up-regulated in lesional skin of AD. IL-36β promoted VEGF-A production via ERK1/2 pathway and PlGF mRNA expression by keratinocytes. IL-36β expression levels in epidermal keratinocytes were correlated with the number of dermal vessels in AD skin. Thus, IL-36β may play an important role for angiogenesis in lesional skin of AD and suppressing IL-36β signaling or expression by spesolimab or dupilumab may be associated with their effectiveness in reducing AD symptoms.

## 4. Materials and Methods

### 4.1. Patients and Tissue Samples

Serum samples were obtained from 36 Japanese patients with AD and 15 healthy control subjects. The characteristics of AD patients and healthy controls are summarized in Table 1. In seven AD patients, serum samples three to six months after dupilumab administration were also collected. Skin samples for immunohistochemistry included lesional skin of AD (*n* = 10) and healthy skin (*n* = 10). The healthy controls had no history of allergy and AD. All patients with AD were enrolled according to the criteria of Hanifin and Rajka [46]. The disease severity was determined by EASI scores. Patients with EASI of less than 7 were considered to be mild and those of more than 21 were considered to be severe. Due to the limited number of mild cases, mild and moderate AD patients were combined in the statistical analysis. All samples were collected during daily clinical practice in St. Marianna University School of Medicine Hospital. The medical ethics committee of St. Marianna University School of Medicine approved all described studies and the study was conducted according to the principles of the Declaration of Helsinki. Written informed consent was obtained to use blood samples from patients and healthy controls. For skin samples, informed consent was obtained in the form of opt-out.

### 4.2. Cell Line

HaCaT cells (kindly provided by Dr Toshio Kuroki, Institute of Molecular Oncology, Showa University, Tokyo, Japan) were cultured in Eagle’s minimum essential medium (Sigma-Aldrich, St Louis, MO, USA) containing 10% fetal bovine serum, penicillin G sodium, streptomycin sulfate, and amphotericin B. When 80% confluence was achieved, the cells were trypsinized, washed, and resuspended in the medium at 1 × 10^6^ cells/mL, and 1 mL was added to each well of the 6-well plates (Becton Dickinson Labware, Franklin Lakes, NJ, USA). When the cells reached confluence, the medium was completely removed and 1 mL serum-free medium was added to each well. Simultaneously, the recombinant human IL-36β protein (R&D systems, Minneapolis, MN, USA) were added, and the cells were incubated at 37 °C and 5% CO_2_. In some experiments, U0126 (MEK1/2 inhibitor; Cell Signaling Technology, Danvers, MA, USA), SB203580 (p38 MAPK inhibitor; Cell Signaling Technology), LY294002 (PI3K inhibitor; Cell Signaling Technology) and sc-514 (IKKβ inhibitor; Abcam, Cambridge, UK), were used. After the indicated time points, cell culture supernatants, and total mRNA were collected for further studies.

### 4.3. RNA Isolation and Quantitative Reverse Transcription-PCR

Total mRNA was obtained from cells with RNeasy Mini Kit (QIAGEN, Hilden, Germany). Complementary DNA was synthesized using ReverTra Ace qPCR RT Master Mix (TOYOBO, Osaka, Japan). The mRNA levels were analyzed using quantitative RT-PCR with THUNDERBIRD SYBR qPCR Mix (TOYOBO). The mRNA levels were normalized to those of the GAPDH gene. The relative change in the levels of genes of interest was determined by the 2^–ΔΔCT^ method. Primers are listed in Table 2.

### 4.4. Immunohistochemistry

We performed immunohistochemical staining for IL-36α, IL-36β, IL-36γ, IL-36R, cytokeratin, VEGF-A, PlGF, and CD34 using lesional skin of AD (*n* = 10) and healthy skin (*n* = 10). These sections were stained with rabbit anti-human IL-36α monoclonal antibody (Abcam), rabbit anti-human IL-36β polyclonal antibody (Merck, Darmstadt, Germany), rabbit anti-human IL-36γ polyclonal antibody (Abcam), rabbit anti-human IL-36R polyclonal antibody (Abcam), mouse anti-human cytokeratin (high molecular weight) monoclonal antibody (DAKO, Glostrup, Denmark), goat anti-human VEGF-A polyclonal antibody (R&D systems), rabbit anti-human PlGF polyclonal antibody (Thermo Fisher Scientific, Waltham, MA, USA), mouse anti-human CD34 monoclonal antibody (clone NU-4A1; Nichirei Biosciences, Tokyo, Japan) or control antibodies followed by ABC staining (Vector Lab, Burlingame, CA, USA). Diaminobenzidine or alkaline phosphatase was used for visualizing the staining, and counterstaining with Mayer hematoxylin was performed, according to manufacturer’s instructions. The double immunohistochemical staining of IL-36β and cytokeratin or CD34 was conducted using ImmPRESS Duet Double Staining Polymer Kit (VectorLabs, Burlingame, CA, USA). The IL-36β immunostaining intensity in all AD and healthy skin samples was evaluated independently by two blinded investigators according to the following scores: score 0 (absent), score 1 (weak expression), score 2 (moderate expression), score 3 (strong expression), and score 4 (very strong expression). The number of CD34^+^ vessels was counted in three low-power fields and the average number was calculated.

### 4.5. Enzyme-Linked Immunosorbent Assay

Serum IL-36β levels and supernatant levels of VEGF-A and PlGF were quantified using the human IL-36β/IL-1F8 DuoSet ELISA (R&D systems), human VEGF Quantikine ELISA Kit (R&D systems), and human PlGF Quantikine ELISA Kit (R&D systems) according to the manufacturer’s instructions. These assays employ the quantitative sandwich enzyme immunoassay technique.

### 4.6. Statistics

Statistical analysis between two groups was performed using the Mann–Whitney U test. The Steel–Dwass test was used for multiple group comparisons. A paired t test was used to determine significant differences in serum IL-36β levels before and after dupilumab treatment. Correlation coefficients were determined by using Spearman’s rank correlation test. *p* values of <0.05 were considered statistically significant.

## Figures and Tables

**Table 1:** Characteristics of atopic dermatitis patients and healthy controls.

	Atopic Dermatitis (*n* = 36)	Healthy Controls (*n* = 15)
Sex (Male:Female)	26:10	8:7
Age (years)	38.9 ± 12.4	44.3 ± 9.6
Atopic comorbidities		
Allergic rhinitis	12 (33.3%)	0 (0%)
Allergic conjunctivitis	1 (2.6%)	0 (0%)
Asthma	8 (22.2%)	0 (0%)
EASI	20.6 ± 10.1	-
The number of eosinophils in peripheral blood (/μL)	484 ± 397	-
Serum LDH levels (U/L)	259 ± 114	-
Serum IgE levels (IU/mL)	10,628 ± 9808	-
Serum TARC levels (pg/mL)	3487 ± 5279	-

Continuous variables are described as the mean ± standard deviation. Abbreviations: EASI, Eczema Area and Severity Index; LDH, lactate dehydrogenase; TARC, thymus and regulated chemokine.

**Table 2:** List of primers.

Target	Forward/Reverse (5′-3′)
IL-25	CCAGGTGGTTGCATTCTTGG/TGGCTGTAGGTGTGGGTTCC
IL-33	GGAAGAACACAGCAAGCAAAGCCT/ TAAGGCCAGAGCGGAGCTTCATAA
TSLP	CCCAGGCTATTCGGAAACTCA/ACGCCACAATCCTTGTAATTGTG
CCL17	GCAAAGCCTTGAGAGGTCTTTGA/CGGTGGAGGTCCCAGGTAGT
CCL26	TCTGTACCCATCCAAGGAAAA/GGGTCCATGTAGCCTTCAGA
VEGF-A	AGCCTTGCCTTGCTGCTCTAC/TCCTCCTTCTGCCATGGGT
PlGF	GCGATGAGAATCTGCACTCTGT/TCCCCAGAACGGATCTTTAGG
GAPDH	ACCCACTCCTCCACCTTTGA/CATACCAGGAAATGAGCTTGACAA

Abbreviations: GAPDH, glyceraldehyde-3-phosphate dehydrogenase; PlGF, placental growth factor; TSLP, thymic stromal lymphopoietin; VEGF-A, vascular endothelial growth factor A.

## Data Availability

The data that support the findings of this study are available from the corresponding author, T.M., upon reasonable request.

## Supplementary Materials

The following supporting information can be downloaded at: https://www.mdpi.com/article/10.3390/ijms241311104/s1.
