# Supplementary material for: Increased Interleukin-36β Expression Promotes Angiogenesis in Japanese Atopic Dermatitis
Source: Int J Mol Sci. 2023 Jul 5;24(13):11104. doi: 10.3390/ijms241311104 (PMC10341928; doi:10.3390/ijms241311104)
Supplement: Supplementary file 1 [file ijms-24-11104-s001.zip › ijms-2394030-supplementary.pdf]

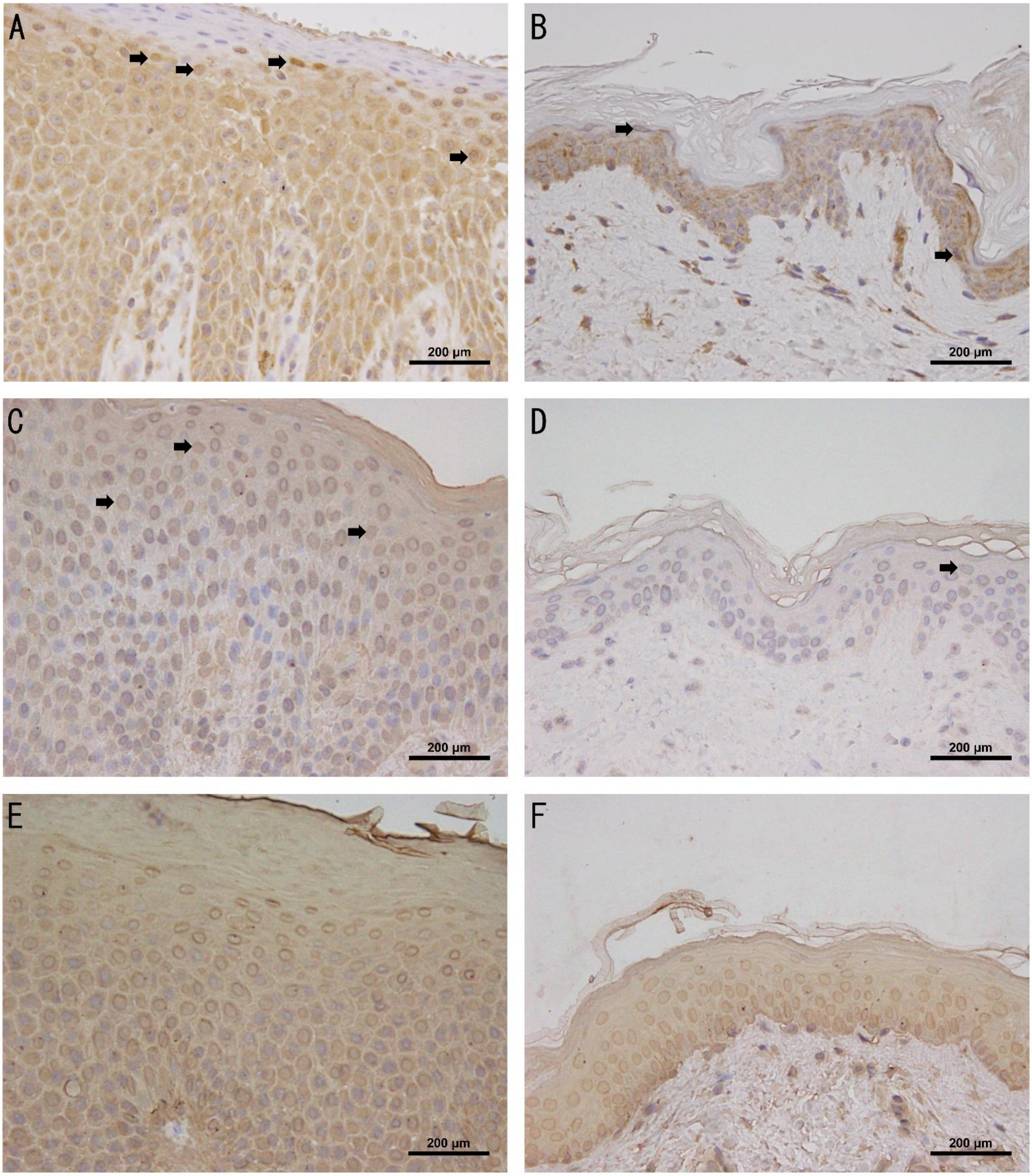

Supplementary Figure S1. IL-36α and IL-36γ is overexpressed on epidermal keratinocytes in lesional skin of atopic dermatitis (AD) and IL-36 receptor (IL-36R) expression on epidermal keratinocytes was comparable between lesional skin of AD and healthy skin. (**A-F**) IL-36α (**A, B**), IL-36γ (**C, D**), and IL-36R (**E, F**) staining in healthy skin and AD lesional skin (n = 10, respectively). Representative images of IL-36α, IL-36γ, and IL-36R staining in AD skin (**A, C, E**) and in healthy skin (**B, D, F**) are shown (Original magnification ×400). Arrows point to representative keratinocytes with the nuclear staining.
